# Confirmatory factor analysis and measurement invariance of the English, Mandarin, and Malay versions of the SF-12v2 within a representative sample of the multi-ethnic Singapore population

**DOI:** 10.1186/s12955-021-01709-9

**Published:** 2021-03-10

**Authors:** Jue Hua Lau, Edimansyah Abdin, Janhavi Ajit Vaingankar, Saleha Shafie, Rajeswari Sambasivam, Shazana Shahwan, Julian Thumboo, Siow Ann Chong, Mythily Subramaniam

**Affiliations:** 1 Research Division, Institute of Mental Health, Buangkok Green Medical Park, 10 Buangkok View, Singapore, 539747 Singapore; 2 Department of Rheumatology and Immunology, Singapore General Hospital, SingHealth, Singapore, Singapore

**Keywords:** SF-12v2, Cross-sectional, Singapore population, Validity, Measurement invariance

## Abstract

**Background:**

The Short Form Health Survey (SF-12v2) is an increasingly popular measure of health-related quality of life (HRQoL) in Singapore. In order to examine whether the SF-12v2 was appropriate for use in the population, the factor structure and validity of the English, Mandarin, and Malay versions were assessed in a representative sample of the general population of Singapore.

**Methods:**

6126 respondents were recruited for the Singapore Mental Health Study 2016 (SMHS 2016), a cross-sectional and population-based survey. Confirmatory factor analyses (CFA) were conducted to examine the fit of a two-factor model for the SF-12v2 within a representative sample and amongst the different language (English, Mandarin, Malay) subgroups. Multiple-group CFAs (MGCFA) were conducted to test measurement invariance across the different languages, ethnicities, and chronic illnesses subgroups. CFA-generated latent factor scores (FSCORE command in MPlus) were also compared with the composite scores derived from the developer’s scoring method via correlations. Sociodemographic correlates of the latent physical and mental health scores were explored.

**Results:**

CFA results within the full sample supported a two-factor model (RMSEA = 0.044; CFI = 0.991; TLI = 0.988; SRMR = 0.044) in which physical functioning, role physical, bodily pain and general health items loaded onto a latent physical health factor, while role emotional, mental health, social functioning, and vitality items loaded onto a latent mental health factor. Physical and mental health factors were allowed to correlate, unlike the developer’s orthogonal scoring method. All standardized loadings were high and statistically significant. Both factors had high internal consistency. CFA within subsamples of English, Mandarin, and Malay languages indicated similar findings. MGCFA results indicate that measurement invariance held across the different languages, ethnicities, and those with and without chronic illnesses.

**Conclusion:**

The present study identified a two-factor (physical and mental health) structure within the general population and amongst the three different languages and demonstrated the measurement invariance of SF-12v2 across different subgroups. Findings indicate that algorithm-derived PCS and MCS should be interpreted with caution as they may result in inaccurate conclusions regarding the relationships between HRQoL and its correlates. Future studies using the SF-12v2 within the general population of Singapore should consider utilizing the factor structure put forth in the present study to obtain more appropriate estimates of HRQoL.

**Supplementary Information:**

The online version contains supplementary material available at 10.1186/s12955-021-01709-9.

## Background

Self-reported measures of health-related quality of life (HRQoL) are important for clinicians and researchers to monitor and assess health outcomes at the individual and population levels [3]. They also allow public health policymakers and planners to determine physical and mental health status across different demographic groups, evaluate the effectiveness of healthcare, determine the burden of preventable diseases, injuries, and disabilities, and identify areas for resource allocation. The Short Form Health Survey-version 2 (SF-12v2) is one example of a HRQoL measure that assesses physical and mental health. Developed from a subset of the original SF-36 scale, the SF-12v2 provides a shorter alternative for researchers to reduce burden of completion in large population health surveys that also replicates the summary scores of the SF-36 [54]. The scoring method provided by its developers was based on principle component analysis (PCA) with orthogonal factor rotations and allows for the 12-items to produce two summary scores: Physical Component Summary (PCS) and Mental Component Summary (MCS). This scoring method assumes that these two summary measures are uncorrelated, and that every item contributes some variance to both the PCS and MCS. However, Vilagut et al. [52] state that this scoring method may not optimize the information contained within the items. Similarly, Jakobsson et al. [19] also cautioned against using the scoring algorithm as it assumes that physical and mental health are uncorrelated, which can result in misleading conclusions. Although Ware and Koskinski (2001) state that ‘results based on summary measures should be thoroughly compared with the SF-36 profile before drawing conclusions’, this recommendation is not possible if the SF-36 was not utilized. The crux of the issue is that a shorter and simpler battery of questions that provides the same information can reduce time and energy spent by both respondents and interviewers, and decrease the chances of missing data (due to the smaller number of questions needed to be answered) [39], and therefore researchers may still opt to utilize the SF-12v2 instead of the SF-36.

Singapore is a multi-ethnic Southeast Asian country of approximately 4 million residents (citizens and permanent residents) comprising 74.4% Chinese, 13.4% Malays, and 9.0% Indians, and other ethnic groups (3.2%) [5]. The SF-12v2 has been utilized for research within Singapore amongst different populations and samples: patients with peritoneal dialysis [14, 58], arthritis [17], mental illness [51], substance and alcohol use disorders [26, 59], older adults [57], and the general population [28, 47]. It appears that the usage of the SF-12v2 within Singapore will continue to increase, and hence there is a need to examine its factor structure within the general population. Researchers in other countries have examined the factor structure of the SF-12v2 via exploratory factor analysis or confirmatory factor analysis (CFA) in general and patient populations [4, 19, 21, 22, 37, 41]. Although some support the usage of the SF-12v2 composite scores [21, 37], the factor structures appear to differ across different studies (i.e. item loadings and residuals). In contrast, researchers have also put forth evidence that the interpretation of the composite scores may be limited in different language versions [19, 22]. The factor structure of the SF-12v2 within the general population and multi-ethnic groups has yet to be established within Singapore. A study by Tan et al. [47] replicated the method by which the developers generated the SF-12v2, and suggested that five items of the SF-12v2 should be modified and replaced due to cultural and language issues faced by Singaporean respondents.

In order to make valid comparisons between groups of different characteristics, the measurement model of a health instrument should be invariant (equal across groups), indicating that these characteristics that are unrelated to the measured construct do not affect the item scores [13]. Essentially, examining the measurement invariance of the SF-12v2 allows researchers to assess whether the questionnaire measures latent variables in the same manner for respondents, or measures them differently for different subpopulations [32]. This is an important step in assessing the psychometric properties of an instrument as it allows a researcher to assess whether the observed differences in scores are contaminated by artifacts of measurement or a lack of measurement invariance [10]. An instrument with poor measurement invariance may cause incorrect interpretations regarding findings from different groups. A growing number of studies have assessed measurement invariance of the SF-12v2 across different demographic groups. For example, Galenkamp et al. [12] examined measurement invariance of the SF-12v2 in a multi-ethnic cohort study in Amsterdam, across age, gender, education levels, and ethnic groups. Similarly, a recent study by Ursenbach et al. [50] explored its measurement invariance across gender and rural/remote dwelling groups within a sample of caregivers of patients with cognitive concerns. Although there are other methods for assessing measurement invariance (e.g. item response theory, multiple indicator multiple cause modelling), multiple-group confirmatory factor analyses (CFA) has been the method of choice due to it being an extension of CFA, and the ability to conduct it with ordinal/categorical data [38].

The aims of the present study are fourfold: i) to examine the factor structure of the SF-12v2 within the general population of Singapore by employing a series of CFAs in a representative sample, and amongst the three different language groups (English, Mandarin, and Malay), ii) to assess measurement invariance across the different language, ethnic (Chinese, Malay, Indian, Others) and chronic physical condition (none, at least one) groups via MGCFA; iii) to compare the latent mental and physical health factor scores generated from the most optimal CFA solution (via FSCORE option in MPLUS) with algorithm-derived SF-12v2 composite scores via correlations and determine how closely both sets of scores were correlated with each other, and iv) to explore the sociodemographic correlates of the CFA-derived latent factor scores.

## Methods

### Study procedure and participants

The present dataset was obtained from the Singapore Mental Health Study 2016 (SMHS 2016); [45], a population based, cross-sectional epidemiological study of residents in Singapore aged 18 years and above. The SMHS 2016 dataset was based upon a national population registry database of all citizens and permanent residents within Singapore. The sample was randomly selected via disproportionate stratified sampling design according to ethnicity (Chinese, Malay, Indian, Others), and age groups (18–34, 35–49, 50–64, 65 and above). The study oversampled certain minority populations, such as residents aged 65 and above, and those of Malay and Indian ethnicities to ensure sufficient sample size and to improve the reliability of the parameter estimates for these population subgroups. Selected residents in the database were sent notification letters followed by a personal home visit by a trained interviewer from a survey research company to obtain their agreement to participate in the study. Face-to-face interviews were conducted in either English, Mandarin, or Malay (the language the respondent was most comfortable with) with those who agreed to participate. Residents who were incapable of doing an interview due to severe physical or mental conditions, language barriers, institutionalization or hospitalization at the time of survey, were living outside of the country, and were unable to be contacted due to incomplete or incorrect addresses, were excluded from the survey. A total number of 6126 respondents were interviewed, with a response rate of 69.5%.

### Measures

The Short Form Health Survey version 2 (SF-12v2) is a 12-item questionnaire that was developed from the 36-item Short Form Health Survey (SF-36), which is a widely used self-report instrument to assess HRQoL amongst patient populations [54]. Similar to the SF-36, the SF-12v2 is a shorter alternative and covers eight sub-domains: general health (GH), physical functioning (PF), role physical (RP), bodily pain (BP), vitality (VT), social functioning (SF), role emotional (RE), and mental health (MH). Items and their respective codes can be found in Table 3. The 12-items that comprise these subdomains are weighted and summarized into two composite summary scores using a scoring algorithm provided by its developers—a physical component summary score (PCS) and a mental component summary score (MCS) which reflect physical and emotional health-related QOL respectively. Based on the theoretical test model, the GH, PF, RP, and BP primarily comprise the PCS, while the RE, MH, VT, and SF subdomains primarily comprise the MCS [37, 41]. Both summary scores range from 0 to 100, with higher scores indicating better HRQoL. The SF-12v2 in the present study was administered in three languages (English, Mandarin, and Malay) that were licensed and translated by the developers. The SF-12v2 was administered via computer-assisted personal interviewing, in which respondents answered questions posed to them via interviewers in their preferred language, and their answers were recorded onto an electronic device. Prior to the commencement of data collection, cognitive interviews and pre-testing were conducted with lay members of the general public in each of the specified languages in order to assess whether the different versions were understood in the manner intended and to identify items that were misinterpreted. These cognitive interviews indicated that the language versions of SF-12v2 were appropriate for use in the population.

Information regarding the chronic physical conditions of the PWD was collected using a chronic conditions checklist [1]. Respondents were asked whether they had any of the following chronic physical conditions: asthma; diabetes mellitus; hypertension or high blood pressure; chronic pain (arthritis or rheumatism, back problems including disc or spine problem, migraine headaches); cancer; cardiovascular disorders (stroke or major paralysis, heart attack, coronary heart disease, angina, congestive heart failure or other heart disease); ulcer, chronic inflamed bowel disease, enteritis or colitis; thyroid disease, neurological conditions, and chronic lung disease. Responses to the checklist were then grouped into two groups: “no chronic physical conditions”, “at least one chronic physical condition”.

Respondents were asked to rate their perceived overall physical and mental health via two questions: (i) “How would you rate your overall physical health” and (ii) “How would you rate your overall mental health”. Each item was scored on a 5-point Likert scale (1 = excellent, 5 = poor), with higher scores indicating poorer health.

Sociodemographic information such as age, gender, and ethnicity were also collected.

### Statistical analysis

Analysis in the present study were conducted with MPlus version 8.2 and Stata version 15. All factor analyses and regression analyses apart from CFAs conducted within the English, Mandarin, and Malay language subsamples were weighted using survey weights to account for complex survey design.

#### Confirmatory factor analyses: factor structure

CFA was utilized to evaluate the factor structure of the SF-12v2 amongst a nationally representative sample of Singapore. As the 12-items of the SF-12v2 were measured on an ordinal scale, a weighted-least-squares with mean- and variance-adjusted (WLSMV option in MPlus) estimation was used to model the polychoric correlation matrix. The following fit indices were utilized to compare the overall model fit and complexities of the models: (i) root mean square error of approximation (RMSEA), (ii) comparative fit index (CFI), (iii) Tucker-Lewis index (TLI), (iv) Standardized Root Mean Square Residual (SRMR). Both CFI and TLI values range from 0 to 1, with higher values representing better fit. CFI values above 0.95 and TLI values above 0.90 are considered to be of excellent fit [23]. With regards to RMSEA, values below 0.08 indicate moderate fit, while values of 0.05 or less indicate close fit to the observed data [18]. Standardized root mean squared residual (SRMR) values were also evaluated, which indicates acceptable fit when values are smaller than 0.08, and good fit when values are smaller than 0.05 [18, 23].

For the full sample, three measurement models were tested using complex survey data (STRATA and WEIGHT options in MPlus) to investigate the factor structure of the SF-12v2. First, a unidimensional model (Model 1) in which all items were specified to load onto a single latent factor. Second, a two-factor model (Model 2) based upon the measurement models suggested in Okonkwo et al. [37] and Shah et al. [41] within which the RP, PF, BP and GH items loaded onto a latent physical health factor, while the RE, VT, MH, and SF items were loaded onto a mental health factor. Thirdly, a two-factor model (Model 3) that was similar to that of Model 2, the difference being that the following residuals of items were allowed to correlate: (i) PF02 and PF04, (ii) RP02 and RP03, (iii) RE02 and RE03, (iv) MH03 and VT02, and (v) MH03 and MH04. CFAs in previous studies on the SF-12v2 have allowed for these correlated residuals [8, 30, 31, 37, 41, 55]. In both Models 2 and 3, the latent physical and mental factors were allowed to correlate. Nested model comparisons were conducted to examine the incremental fit of Model 2 over Model 1, and Model 3 over Model 2. Chi-square difference tests (DIFFTEST option in MPlus) with the WLSMV estimation was utilized to examine the statistical significance of any improvements in fit between the models. After comparing the fit indices and results from the nested model comparisons, the best fitting model was chosen. Internal consistency was evaluated using composite reliability values for the best fitting model for the full sample, where the acceptable level was set at 0.70 [15].

In order to determine whether the factor structure was consistent across different languages and had acceptable fit to proceed with a further MGCFA, a subgroup analysis was conducted. The full sample was split into three subsamples based on the language the survey was conducted in: English, Mandarin, and Malay. In each subsample, Models 2 and 3 were estimated without any sampling weights. Fit indices and factor solutions of the two models in each subsample were compared with Chi-square difference tests (DIFFTEST option in Mplus).

#### Multiple-group confirmatory factor analyses: measurement invariance

In order to examine measurement invariance (i.e. whether relationships between responses to items and latent constructs were the same across groups), a multiple group CFA was conducted across the three languages (English, Mandarin, Malay), and four ethnic groups (Chinese, Malay, Indian, Others), and chronic physical conditions (None, At least one) using complex survey data. A series of nested multiple-group CFA models with increasing parameter constraints were conducted to test for three hierarchic levels of measurement invariance as per recommendations of extant literature [13, 38]. Firstly, configural invariance was examined in a model where the factorial structure was invariant across groups with no equality constraints imposed, relying on common model fit indices. This assesses whether clustering of items and their respective factors do not vary across groups. Secondly, metric invariance was tested by fitting models where factor loadings on respective items were constrained to be equal across groups. This examines whether the magnitude of relationships between items and factors are equivalent across groups. Third, scalar invariance was tested by additionally constraining thresholds to be equal across groups, in order to examine whether item thresholds were comparable across groups. Extant literature has recommended the use of two metrics by which to evaluative model fit: change in CFI (Δ CFI) and RMSEA (Δ RMSEA). These are obtained by comparing the fit indices between more and less constrained models. Following the recommendations of Rutkowski and Svetina [40] for MGCFA with categorical indicators, a measure can be considered invariant at a particular level if the Δ CFI does not decrease by > 0.004, ΔRMSEA does not increase by > 0.05 for metric invariance and > 0.01 for scalar invariance. For the MGCFA examining measurement invariance across language groups only, item MH04 was subsumed into four categories: “All/Most of the time”, “Some of the time”, “A little of the time” and “None of the time”. This was because no respondents in the Mandarin language group endorsed “All of the time” to this item which led to an error when running the MGCFA on MPlus. Items were not transformed for the other MGCFAs.

#### Correlation: comparing latent factor scores with algorithm-generated composite scores

Using the FSCORE command within MPlus physical and mental health latent factor scores were generated for subsequent analyses. This method of obtaining factor scores over merely summing scores on respective items allows for the adjustment for measurement error and therefore provides better power and measurement reliability [23]. Items GH01, BP02, MH03, and VT02 were not reversed in the CFA model and were thus unreversed in the calculation of latent factor scores. Pearson correlations were then calculated between the physical and mental health latent factor scores, and the SF-12v2 algorithm-generated composite scores in order to determine whether the algorithm-generated scores closely resembled the latent factor scores.

#### Linear regression: sociodemographic correlates of physical and mental health factor scores

Two linear regressions were conducted to explore the associations between sociodemographic factors, perceived overall physical health, perceived overall mental health and the latent physical and mental factor scores.

## Results

### Sociodemographic characteristics of the sample

A total of 6126 respondents participated in the study. Weighted and unweighted frequencies regarding sociodemographic information of the sample are displayed in Table 1. Based on weighted frequencies, 50.4% were female, and 75.7% were of Chinese ethnicity. 75.6%  completed the survey in the English language, while 21.2% completed the survey in Mandarin, and 3.1%  completed the survey in Malay. Seven (0.11%) participants did not complete the SF-12v2 and were thus excluded from analyses. 6 cases (2 English and 4 Malay language) had missing data on the SF-12v2 and were further excluded listwise from the analyses. 71.7%, 78.7%, 97.7%, and 94.2% of participants from the Chinese, Malay, Indian, and Other ethnicity groups respectively, chose English language for the study.

**Table 1:** Sociodemographic characteristics of the sample (n = 6126), and crosstabulation of language by ethnicity

Variable	Category	n	Unweighted %	Weighted %
Age	18 to 34	1707	27.9	30.4
	35 to 49	1496	24.4	29.6
	50 to 64	1626	26.5	26.9
	65 and above	1297	21.2	13.1
Gender	Male	3068	50.1	49.6
	Female	3058	49.9	50.4
Ethnicity	Chinese	1782	29.1	75.7
	Malay	1990	32.5	12.5
	Indian	1844	30.1	8.7
	Others	510	8.3	3.1
Language	English	4916	80.3	75.6
	Mandarin	557	9.1	21.2
	Malay	646	10.6	3.1
	Missing^a^	7	0.1	0.1
Chronic Physical Conditions	None	2552	41.7	46.1
	At least one	3562	58.2	53.7
	Missing	12	0.2	0.1

	Language, n (weighted %)			
	English	Mandarin	Malay	Missing
Ethnicity				
Chinese	1230 (71.7%)	546 (27.9%)	4 (0.2%)	2 (0.1%)
Malay	1420 (78.7%)	0	566 (21.2%)	4 (0.1%)
Indian	1782 (97.7%)	0	61 (2.2%)	1 (0.04%)
Others	484 (94.2%)	11 (2.6%)	15 (3.2%)	0

^a^Participants in this group did not answer any of the questions on the SF-12v2

### Confirmatory factor analysis of the full sample

Fit indices for each solution for the full sample and language subsamples, as well as DIFFTESTs and change in fit indices when comparing the models are displayed in Table 2. The CFA results indicated that Model 1, with all items loading onto a single latent factor, had a poor overall fit to the observed data. Comparatively, Model 2 provided a decent fit, with the nested comparison revealing that Model 2 fit significantly better than Model 1. However, the SRMR and RMSEA values indicate that some improvements in fit might still be possible. On the other hand, fit indices of Model 3 indicated excellent fit to the observed, with nested comparison displaying a significantly better fit than compared to Model 2. The standardized factor loadings of Model 3 are presented in Table 3. The physical and mental health latent factors had high internal consistency, with composite reliability values of 0.91 and 0.85 respectively.

**Table 2:** Model indices for confirmatory factor analyses, and measurement invariance across language, ethnicity and chronic conditions

	WLSMV χ^2^ (df)	RMSEA	CFI	TLI	SRMR	DIFF TEST χ^2^ (df), *p*	ΔRMSEA^a^	ΔCFI^a^	ΔTLI	ΔSRMR
*Confirmatory factor analyses*										
Model 1 Unidimensional	3994.848 (54)	0.109	0.940	0.926	0.116	–	–	–	–	–
Model 2 Two–factor model	2304.272 (53)	0.083	0.965	0.957	0.082	554.73 (1), *p* < 0.001	− 0.026	+ 0.025	+ 0.031	− 0.034
Model 3 Two-factor model with correlated residuals	618.043 (48)	0.044	0.991	0.988	0.044	939.031 (5), *p* < 0.001	− 0.039	0.026	+ 0.031	− 0.038
*Confirmatory factor analyses by language subgroups*^b^										
*English language only*										
Model 2 Two-factor model	3652.579 (53)	0.118	0.951	0.939	0.078	–	–	–	–	–
Model 3 Two-factor model with correlated residuals	1210.750 (48)	0.070	0.984	0.978	0.046	1422.088 (5), *p* < 0.001	− 0.048	+ 0.033	+0.039	− 0.032
*Mandarin language only*										
Model 2 Two-factor model	543.064 (53)	0.129	0.962	0.953	0.097	–	–	–	–	–
Model 3 Two-factor model with correlated residuals	206.138 (48)	0.077	0.988	0.983	0.057	208.193 (5), *p* < 0.001	− 0.052	+ 0.026	+ 0.03	− 0.04
*Malay language*										
Model 2 Two-factor model	566.189 (53)	0.123	0.987	0.983	0.074	–	–	–	–	–
Model 3 Two-factor model with correlated residuals	310.606 (48)	0.092	0.993	0.991	0.052	169.298 (5), *p* < 0.001	− 0.025	+ 0.006	+ 0.008	− 0.022
*Measurement invariance (MGCFA)*										
*Language*										
Configural	895.138 (144)	0.051	0.991	0.988	0.046	–	–	–		
Metric	879.544 (164)	0.046	0.991	0.99	0.046	54.333 (20), *p* < 0.001	− 0.005	0		
Scalar	1178.003 (222)	0.046	0.988	0.99	0.05	378.713 (58), *p* < 0.001	0	− 0.003		
*Ethnicity*										
Configural	1473.219 (195)	0.065	0.991	0.988	0.048	–	–	–		
Metric	1354.748 (225)	0.057	0.992	0.991	0.048	33.096 (30), *p* = 0.32	− 0.008	+0.001		
Scalar	1398.609 (312)	0.048	0.992	0.993	0.049	231.257 (87) , *p* < 0.001	− 0.01	0		
*Chronic physical conditions*										
Configural	680.066 (97)	0.044	0.991	0.987	0.047	–	–	–		
Metric	654.824 (107)	0.041	0.991	0.989	0.048	39.467 (10), *p* < 0.001	− 0.003	0		
Scalar	666.124 (136)	0.036	0.991	0.992	0.053	87.085 (29), *p* < 0.001	− 0.005	0		

^a^Only change in RMSEA and CFI were evaluated for MGCFA

^b^The CFAs within the language subgroups were not weighted using survey weights

**Table 3:** Standardized factor loadings for the final two factor model (Model 3) for the SF-12v2 amongst total sample, and subsamples based on questionnaire language

SF-12v2 items	Total sample (n = 6113^a^)	Subsamples split by language		
		English (n = 4914^a^)	Mandarin (n = 557^a^)	Malay (n = 642^a^)
	Estimate	Estimate	Estimate	Estimate
*Latent physical factor*				
General health (GH01)	− 0.54	− 0.58	− 0.64	− 0.55
Limited in moderate activities (PF02)	0.78	0.77	0.73	0.85
Limited in climbing several stairs (PF04)	0.73	0.73	0.68	0.83
Accomplished less due to physical health (RP02)	0.93	0.93	0.93	0.94
Limited in kind of work (RP03)	0.94	0.95	0.90	0.96
Pain interfered with work (BP02)	− 0.74	− 0.73	− 0.73	− 0.81
*Latent mental factor*				
Accomplished less due to emotional problems (RE02)	0.89	0.90	0.91	0.88
Worked less carefully (RE03)	0.89	0.90	0.89	0.88
Felt calm and peaceful (MH03)	− 0.42	− 0.47	− 0.38	− 0.49
Have a lot of energy (VT02)	− 0.56	− 0.59	− 0.56	− 0.65
Felt downhearted and depressed (MH04)	0.55	0.59	0.45	0.59
Physical/emotional health problems interfered with social activities (SF02)	0.83	0.84	0.83	0.83
*Latent factor correlation*				
Latent physical factor with latent mental factor	0.80	0.81	0.85	0.86
*Correlated residuals*				
PF02 with PF04	0.70	0.66	0.66	0.64
RP02 with RP03	0.57	0.53	0.57	0.47
RE02 with RE03	0.75	0.71	0.80	0.89
MH03 with VT02	0.60	0.59	0.59	0.46
MH03 with MH04	− 0.32	− 0.32	− 0.34	− 0.36

^a^After accounting for missing/incomplete data

### Confirmatory factor analysis of subgroups by language

The CFA results in the English language subsample revealed that Model 2 had poor fit to the observed data. Comparatively, Model 3 had a significantly better fit. In the Mandarin language subsample, Model 2 was poor in its ability to represent the data. Based on the nested comparison, Model 3 provided a significantly better representation of the data. For the Malay language subsample, Model 2 provided a poor fit to the data as indicated by a high RMSEA value. Model 3 had a significantly better fit based on the nested comparison. The standardized loadings of Model 3 for each language subsample is displayed in Table 3.

### Multiple-group confirmatory factor analyses across language, ethnicity, and chronic condition groups

The factorial structure of Model 3 derived from the CFA analyses was used to examine measurement invariance across language, ethnicity, and chronic conditions groups. Results of the model indices for measurement invariance across these groups are displayed in Table 2, along with information on the ΔCFI and ΔRMSEA when comparing the configural, metric, and scalar models. MGCFA demonstrated good data fit for configural invariance across the language, ethnicity and chronic conditions groups, indicating that Model 3 was confirmed within these subgroups. Furthermore, the addition of constraints for equal factor loadings (metric invariance) and item thresholds (scalar invariance) did not result in poorer model fit as ΔCFI and ΔRMSEA did not exceed their respective thresholds. The findings confirmed configural, metric, and scalar invariance for Model 3.

### Correlations between latent factors and component scores

Latent factor scores were generated based upon Model 3 conducted within the full sample. Pearson correlations were conducted between the latent factor scores and component scores, with coefficients displayed in Table 4. The physical health latent score was highly correlated with the algorithm-scored PCS (r = 0.77, *p* < 0.001) and weakly correlated with MCS (r = 0.39, *p* < 0.001). The mental health latent score was highly correlated with MCS score (r = 0.71, *p* < 0.001) and moderately correlated with PCS (r = 0.52, *p* < 0.001). While the PCS and MCS scores were weakly correlated as expected due to the nature of the orthogonal factor structure employed by its developers, interestingly, this correlation was negative (r = -0.09, *p* < 0.001). As opposed to the PCS and MCS, the physical health and mental health latent factors were strongly correlated with each other (r = 0.90, p < 0.001).

**Table 4:** Unweighted correlations between physical and mental health latent factors and SF-12v2 composite scores

	Physical health latent factor	Mental health latent factor	Physical component score	Mental component score
Physical health latent factor	1.00			
Mental health latent factor	0.90	1.00		
Physical component score	0.77	0.52	1.00	
Mental component score	0.39	0.71	− 0.09	1.00

All pairwise correlations were statistically significant

### Correlates of CFA-derived physical and mental health latent scores

Results of the weighted regression analyses examining the correlates of physical and mental health latent scores are presented in Additional file 1. Results indicate that compared to those aged 18 to 34, respondents who were aged 35 to 49 had higher physical health latent factor scores (B = 0.08, 95% CI: 0.02, 0.14, *p* = 0.01), while those who were aged 65 and above had lower physical health latent factor scores (B = − 0.30, 95% CI: − 0.38, − 0.22, *p* < 0.001). However, there was no significant difference in scores between those aged 18 to 34 and those aged 50 to 64 (*p* = 0.921). Pairwise comparisons also indicated that those aged 65 and above had poorer physical health than those aged 35 to 49 (B = − 0.38, 95% CI: − 0.46, − 0.31, *p* < 0.001), and those aged 50 to 64 (B = − 0.31, 95% CI: − 0.39, − 0.23, *p* < 0.001). Females also had lower physical health latent factor scores (B = − 0.10, 95% CI: − 0.15, − 0.05, *p* < 0.001) than males. Compared to those of Chinese ethnicity, individuals of Indian (B = − 0.10, 95% CI: − 0.15, − 0.05 *p* < 0.001) and Malay (B = − 0.05, 95% CI: − 0.09, − 0.001, *p *= 0.047) ethnicit had  lower physical health latent factor scores. Pairwise comparison indicated that Indians had poorer physical health than Malays (B = − 0.05, 95% CI : − 0.10, − 0.003, *p* = 0.04). Results also indicated that both poorer perceived overall physical health (B = − 0.27, 95% CI: − 0.30, − 0.23 , *p* < 0.001) and poorer perceived overall mental health (B = − 0.14, 95% CI: − 0.18, − 0.11, *p* < 0.001) were associated with lower physical health latent factor scores.

Results revealed that compared to those aged 18 to 34, individuals aged 35 to 49 (B = 0.18, 95% CI: 0.12, 0.24, *p* < 0.001) and 50 to 64 (B = 0.18, 95% CI: 0.12, 0.25, *p *< 0.001) had higher mental health latent factors scores. However, there was no difference between those aged 18 to 34 and those aged 65 and above (p = 0.28). Females were also associated with  poorer mental health latent factor scores (B = − 0.09, 95% CI: − 0.14, − 0.04 , *p* < 0.001). Compared to those of Chinese ethnicity, Indians had poorer mental health latent factor scores (B = − 0.06, 95% CI: − 0.10, − 0.01, *p* = 0.02). Poorer perceived overall physical health (B = − 0.19, 95% CI:  − 0.23, − 0.16, *p* < 0.001) and poorer perceived overall mental health (B = − 0.20, 95% CI: − 0.23, − 0.16, *p* < 0.001) were both associated with lower mental health latent factor scores.

## Discussion

### Factor structure of the SF-12v2: correlated physical and mental health factors

The present study is the first to examine the factor structure of the SF-12v2 within the Singapore resident population. Extant literature has identified some limitations in the scoring algorithm for the SF-12v2. One such limitation appears to arise from the orthogonal rotation utilized in its development, in which summary scores are forced to be uncorrelated, and this methodology has been criticized within literature for both the SF-12v2 [7, 19] and SF-36 [46, 49]. Another limitation of the PCS and MCS is the assumption that all items contribute to both composite scores and are either positively or negatively weighted. In essence, this means that higher raw scores on items regarding physical health not only contribute to higher PCS scores, but influence MCS scores negatively [19]. Due to these negative weights and the orthogonal factor analysis they are derived from, the current algorithm-generated scores may be counterintuitive of the underlying raw scores and may present issues during interpretation [7, 9, 35]. This issue was observed in the current study as well. Although weak, it appears that there was a significant negative correlation (− 0.09) between the algorithm-based composite scores within the present sample, and this finding therefore questions the validity of the SF-12v2 summary measures. Furthermore, Maurischat et al. [30] posited that both physical and mental health measured by the SF-12v2 appear to not be independent, as the responses on items are influenced by similar wordings on the questions. Other researchers in the field of HRQoL have also suggested that both physical and mental health are not independent of one another [29, 36]. This assumption is similarly supported in the present study, which found a strong intercorrelation between the latent factors in the final model (0.90), providing further evidence that both constructs are strongly and positively related, and is counter to the algorithm-based scores which were weakly and negatively correlated. The validity of the factor structure within the present study was tested by examining models by Shah et al. [41], Okonkwo et al. [37], and Noor and Aziz [34], and  did not allow for items to cross load on factors, but allowed for covariance between factors. Based on the excellent fit of final model in the present data, it is strongly suggested that allowing for the covariance between the latent factors would be an improved method of handling data for the SF-12v2 within the Singaporean population over the standard scoring algorithms.

Some studies have utilized high correlations between the physical and mental latent factors derived from a CFA and the composite scores as evidence to support to use of the SF-12v2. For example, Okonkwo et al. [37] found that the physical and latent factors had correlations of 0.97 and 0.96 with their respective composite scores. Similarly, Shah et al. [41] found almost perfect correlations between the latent factors and their composite scores. However, this was not the case with the current study. Although the association was strong (0.71–0.77), the latent factor scores were neither perfectly nor close to perfectly correlated with the composite scores, therefore providing evidence that the composite scores do not adequately reflect the scores generated from the final CFA model.

The results of the CFAs within the different language subgroups also demonstrated that the final model was a good fit in both the English and Mandarin language groups and lends further evidence that the original scoring algorithm may not be valid within the Singaporean population. In studies conducted elsewhere, the factor structure of the Mandarin and Malay versions corroborated the present results. For example, a CFA conducted by Su and Wang [44] on a Mandarin version of the SF-12v2 with 1000 older adults in China, was similar to the final model in the present study. Another study utilizing a similar model, using the Malay version of the SF-12v2 in a sample of 108 post-partum mothers in Malaysia found an acceptable fit.

### Measurement invariance of the final model across language, ethnicity, and chronic conditions

Research on cross-cultural perceptions of health has identified that cultural beliefs or expectations may lead to differences in the interpretation of items on a questionnaire [24]. In a Singapore-based study, Tan et al. [47] posited that the item MH04 “During the past 4 weeks, have you felt downhearted and depressed” was inappropriate for measuring mental health among Singaporeans due to it being a taboo topic for fear of discrimination. The present study disagrees with the suggestions of Tan et al. [47] to replace items, since the results of the MGCFA demonstrated that measurement invariance of the final model held across the different language versions, as well as the ethnic and chronic conditions subgroups. Furthermore, the present study also found a good fit of the final model across the different language versions, as well moderate to high loadings of the items amongst the full sample and across languages. These results indicate that the items of the SF-12v2 are interpreted in the same way across groups, and the underlying factor structure of Model 3 is measured in the same manner across respondents and subpopulations. Therefore, the different language versions are appropriate for use within the Singaporean population. To date, no other studies appear to have examined measurement invariance of the SF-12v2 within the Singaporean population across the different language versions. It would be prudent for future studies to replicate the present methodology within representative samples  and subgroups, in order to further examine the validity of the SF-12v2.

The present study suggests that US-based norms should not be utilized when scoring the SF-12. For example, researchers in Hong Kong developed population norms for the Chinese version of the SF-12v2 due to the difference from US population in scores on physical functioning and vitality [25]. Similarly, studies in New Zealand [11] and the Netherlands [33], have established their own population based norms for the SF-12v2. Applying US norm weights will result in different emphasis being placed upon items, and due to cultural differences in the perception of health, will result in inaccurate estimates of the underlying raw scores. Norms and weights for the SF-36 have been already established within the Singaporean population by Sow et al. [43], which was used by Tan et al. [47] to develop the Singapore version of the SF-12v2 (SG-12). However, given that both the weights and development of the SG-12 were not based upon a factor analysis within the Singaporean population, and instead upon the traditional scoring method in which the two factors were forced to be uncorrelated, the authors of the present study argue against its usage. Therefore, the present study suggests that future studies using the SF-12v2 within the Singaporean population should avoid using the traditional scoring method and instead conduct a CFA to generate latent factor scores.

### Correlates of physical and mental health within the singaporean population

As expected, poorer health on the items examining overall physical and mental health were associated with lower scores on the physical and mental health latent factor scores. Women were found to have poorer scores on both physical and mental health. Both physical and mental HRQoL were poorer in Indian participants than Chinese. Leow et al. [27] also had similar findings, in that Indian participants had poorer health scores than Chinese participants, but only on the PCS. On the other hand, Thumboo et al. [48] found that Indians had poorer scores on the SF-36 PCS, and also poorer mental health on subscales of the SF-36 MCS. The nation-wide and cross-sectional SMHS 2016 revealed that Indians were 1.3 times more likely than Chinese to have any mental illness [45]. Furthermore, earlier local research has found that Indians in Singapore were found to be at higher risk of cardiovascular risk factors [2] and diabetes (Singapore Health Promotion [42]. It is plausible that these physical and mental health risks may explain lower physical and mental health amongst this ethnic group. Findings also indicate that Malays had poorer physical health than Chinese. These results suggest that Indians and Malays are vulnerable ethnic groups that may require further support in terms of health outcomes.

The older adult group (i.e. above 65 years) appeared to have poorer physical health than those aged 18 to 34. Perhaps this may be explained by the presence of chronic illnesses, which tends to increase with age [6], which affect perceptions of physical HRQoL. With regards to mental health, participants in the age groups of 34 to 49 and 50 to 64 had better mental HRQoL than those aged 18 to 34. The role of socioeconomic status on health has been well-established [56]. It may be possible that these groups are more financially established, have higher incomes, and have more access to health services to prevent and treat illnesses. On the other hand, there was no significant difference between the older adult group and those younger than 34 years on the mental health score. This lack of a difference in mental health score may indicate that both groups have similarly poorer mental health. In the case of the older adult group, it is plausible that they have lower energy and are more susceptible to fatigue due to chronic health conditions, and thus had lower mental health latent scores. A recent paper by Subramaniam et al. [45] found that compared to other age groups, those within age 18 to 34 had the highest lifetime prevalence of mental disorders, which may indicate why this group had poorer scores on the mental health factor.

### Strengths, limitations and avenues for future research

A strength of the present study is that the sample is nationally representative, and the results are therefore generalizable to the Singapore resident population. Furthermore, the sample size is more than adequate for factor analytic procedures, even for the MGCFAs performed. It would also be interesting for future studies to examine the sensitivity and specificity of the CFA-generated factor scores and obtain optimal cut-off points for the detection of certain disorders [52]. Alternative scoring methods such as the RAND-12 yields physical and mental summary scores based upon oblique factor scores [16]. This approach further differs from the traditional scoring method in that it not only does not apply negative weights, but also, only uses the scores that are presumed to represent their respective summary measures. Studies have suggested that the RAND-12 method appears to perform better than the original SF-12v2 scoring method at identifying differences in health in certain clinical populations [20, 35] due to its oblique factor scores. However, this method uses US population norms established in 1998. It would be interesting for a follow-up study to examine how the RAND-12 scoring method fairs in comparison to the traditional SF-12v2 scoring method, and latent factor scores derived from a CFA.

## Conclusion

In conclusion, the present study identified a two-factor (physical and mental health) factor structure in a representative sample and amongst the three different languages. This study also demonstrated the measurement invariance of SF-12v2 across different languages, ethnicities, and amongst those with or without chronic physical conditions in Singapore. The orthogonal scoring algorithm put forth by the developers assumes that physical and mental health are independent of each other, and this has been criticized in extant literature. Findings of the present study indicate that the algorithm-derived PCS and MCS should be interpreted with caution as they may result in inaccurate conclusions regarding the relationships between HRQoL and its correlates. Therefore, future studies using the SF-12v2 within the general population of Singapore should consider utilizing the factor structure put forth in the present study to obtain more appropriate estimates of physical and mental health.

## Supplementary Information

**Additional file 1.** Results of the Linear Regression Models.

## Data Availability

The datasets used and/or analysed during the current study are available from the corresponding author on reasonable request.

## Abbreviations

HRQOL: Health Related Quality of Life
SF-12v2: 12-Item Short Form Health Survey Version 2
SF-36: 36-Item Short Form Health Survey
PCS: Physical Component Score
GH: General health
PF: Physical functioning
RP: Role physical
BP: Bodily pain
MCS: Mental component score
RE: Role emotional
MH: Mental health
VT: Vitality
SF: Social functioning
PCA: Principal components analysis
CFA: Confirmatory factor analysis
MGCFA: Multiple Group Confirmatory Factor Analysis
WLSMV: Weighted-least-squares with mean- and variance-adjusted estimator
RMSEA: Root mean square error of approximation
CFI: Comparative fit index
TLI: Tucker-Lewis index
SRMR: Standardized root mean square residual
B: Unstandardized regression coefficient
