# Supplementary material for: Confirmatory factor analysis and measurement invariance of the English, Mandarin, and Malay versions of the SF-12v2 within a representative sample of the multi-ethnic Singapore population
Source: Health Qual Life Outcomes. 2021 Mar 10;19:80. doi: 10.1186/s12955-021-01709-9 (PMC7944897; doi:10.1186/s12955-021-01709-9)
Supplement: Supplementary file 1 — Additional file 1. Results of the Linear Regression Models. [file 12955_2021_1709_MOESM1_ESM.docx]

| Supplementary Table 1 | | | | | | | | |
| --- | --- | --- | --- | --- | --- | --- | --- | --- |
| Results of the weighted regression analyses examining correlates of Physical and Mental Health Latent Factors | | | | | | | | |
|  |  | Physical Health Latent Factor^a^ | | |  | Mental Health Latent Factor^b^ | | |
|  | Mean^┼^ | B | 95% CI | *p* | Mean^┼^ | B | 95% CI | *p* |
| Age |  |  |  |  |  |  |  |  |
| 18 to 34 | 0.007 | ref |  |  | -0.106 | ref |  |  |
| 35 to 49 | 0.013 | **0.08** | 0.02 – 0.14 | **0.01** | 0.004 | **0.18** | 0.12 – 0.24 | **< 0.001** |
| 50 to 64 | -0.115 | 0.003 | -0.06 – 0.07 | 0.92 | -0.043 | **0.18** | 0.12 – 0.25 | **< 0.001** |
| 65 and above | -0.483 | **-0.30** | -0.38 – -0.22 | **< 0.001** | -0.242 | 0.04 | -0.03 – 0.12 | 0.27 |
| Gender |  |  |  |  |  |  |  |  |
| Male | -0.028 | ref |  |  | -0.019 | ref |  |  |
| Female | -0.147 | **-0.10** | -0.15 – -0.05 | **< 0.001** | -0.128 | **-0.09** | -0.14 – -0.04 | **< 0.001** |
| Ethnicity |  |  |  |  |  |  |  |  |
| Chinese | -0.091 | ref |  |  | -0.081 | ref |  |  |
| Malay | -0.118 | **-0.05** | -0.09 – -0.001 | **0.047** | -0.089 | -0.01 | -0.06 – 0.04 | 0.66 |
| Indian | -0.088 | **-0.10** | -0.15 – -0.05 | **< 0.001** | -0.053 | **-0.06** | -0.10 – -0.01 | **0.02** |
| Others | 0.089 | -0.02 | -0.10 – 0.05 | 0.57 | -0.096 | 0 | -0.08 – 0.08 | 0.99 |
| Overall physical health item | - | **-0.27** | -0.30 – -0.23 | **< 0.001** | **-** | **-0.19** | -0.23 – -0.16 | **< 0.001** |
| Overall mental health item | - | **-0.14** | -0.18 – -0.11 | **< 0.001** | **-** | **-0.20** | -0.23 – -0.16 | **< 0.001** |
| ^┼^Survey weighted means  ^a^ n = 6115, mean = -0.09 ± 0.76, range: -3.51 – 1.51  ^b^ n = 6115, mean = -0.07 ± 0.74, range: -3.55 – 1.49  B – unstandardized regression coefficient; 95% CI: 95% confidence interval of B  Bold print denotes statistically significant B value | | | | | | | | |
